# A Risk Stratification Model for Metastatic Lymph Nodes of Papillary Thyroid Cancer: A Retrospective Study Based on Sonographic Features

**DOI:** 10.3389/fendo.2022.942569

**Published:** 2022-07-22

**Authors:** Xiaofeng Ni, Shangyan Xu, Weiwei Zhan, Wei Zhou

**Affiliations:** ^1^Department of Ultrasound, Ruijin Hospital, Shanghai Jiao Tong University School of Medicine, Shanghai, China; ^2^Department of Ultrasound, RuiJin Hospital/Lu Wan Branch, Shanghai Jiao Tong University School of Medicine, Shanghai, China

**Keywords:** lymph node, papillary thyroid carcinoma, ultrasonography, metastasis, risk stratification

## Abstract

**Background:**

Papillary thyroid carcinoma (PTC) has a high probability of cervical lymph node (LN) metastasis. The aim of the study was to develop an ultrasound risk stratification model to standardize the diagnosis of metastatic LNs of PTC.

**Methods:**

Patients with suspicious thyroid nodules who underwent US examination and US guided fine-needle aspiration for cervical LNs were retrospectively collected. Univariate and multivariate logistic regression analyses were performed to assess the independent risk factor of metastatic LNs. According to the OR value of correlated indicators in logistic regression analysis, a risk stratification model was established.

**Results:**

A total of 653 LNs were included. The independent risk factors of metastatic LNs were long-axis diameter/short-axis ≤ 2 (OR=1.644), absence of hilum (OR=1.894), hyperechogenicity (OR=5.375), calcifications (OR=6.201), cystic change (OR=71.818), and abnormal flow (OR=3.811) (P<0.05 for all). The risk stratification model and malignancy rate were as follows: 0-2 points, malignancy rate of 10.61%, low suspicion; 3-5 points, malignancy rate of 50.49%, intermediate suspicion, ≥6 points, malignancy rate of 84.81%, high suspicion. The area under the receiver operating characteristic curve for the model was 0.827 (95% CI 0.795-0.859).

**Conclusions:**

Our established risk stratification model can effectively evaluate metastatic LNs in the patients with suspicious thyroid nodules, and it might provide a new strategy choice for clinical practice.

## Introduction

Thyroid cancer is an endocrine neoplasm, and its incidence increases in recent years. Papillary thyroid carcinoma (PTC) is the most common type of thyroid cancer. Although the prognosis of PTC is good, it is prone to present lymph node (LN) metastases (1–3). The metastasis rate of central LN is 20-50%, and the rate of lateral LN is 10-30% (4). Ultrasonography (US) is the most widely used tool for detecting metastatic LNs, which can also effectively guide fine-needle aspiration (FNA) for suspicious LNs (5). However, US has the characteristics of high specificity and low sensitivity for metastatic LNs, especially for LNs in the central region (6, 7).

Previous studies have mostly focused on the sonographic features of metastatic LNs of PTC, but there were few studies about risk stratification. The risk stratification system has been applied in several organs (8–10), which not only can make diagnosis more standardized but also can provide effective guidance for clinical treatment. Ryu et al. had studied the prediction rule model for detecting metastatic LNs of PTC; however, it only included the malignant indicators of LNs, and the risk weight of each indicator was not considered (11). Different indicators of LNs had different odds ratio(OR)value, which implied the different weight of malignancy. In our research, we planned to develop a risk stratification model of cervical LNs in the patients with PTC, which was based on different weights of the indicators.

## Materials and Methods

### Patient Selection

This retrospective study was approved by the Institutional Review Board, and the requirement for written informed consent was waived. Consecutive patients who underwent US examination and US-FNA for cervical LNs at our hospital from January 2017 to December 2019 were collected.

**Figure 1:**
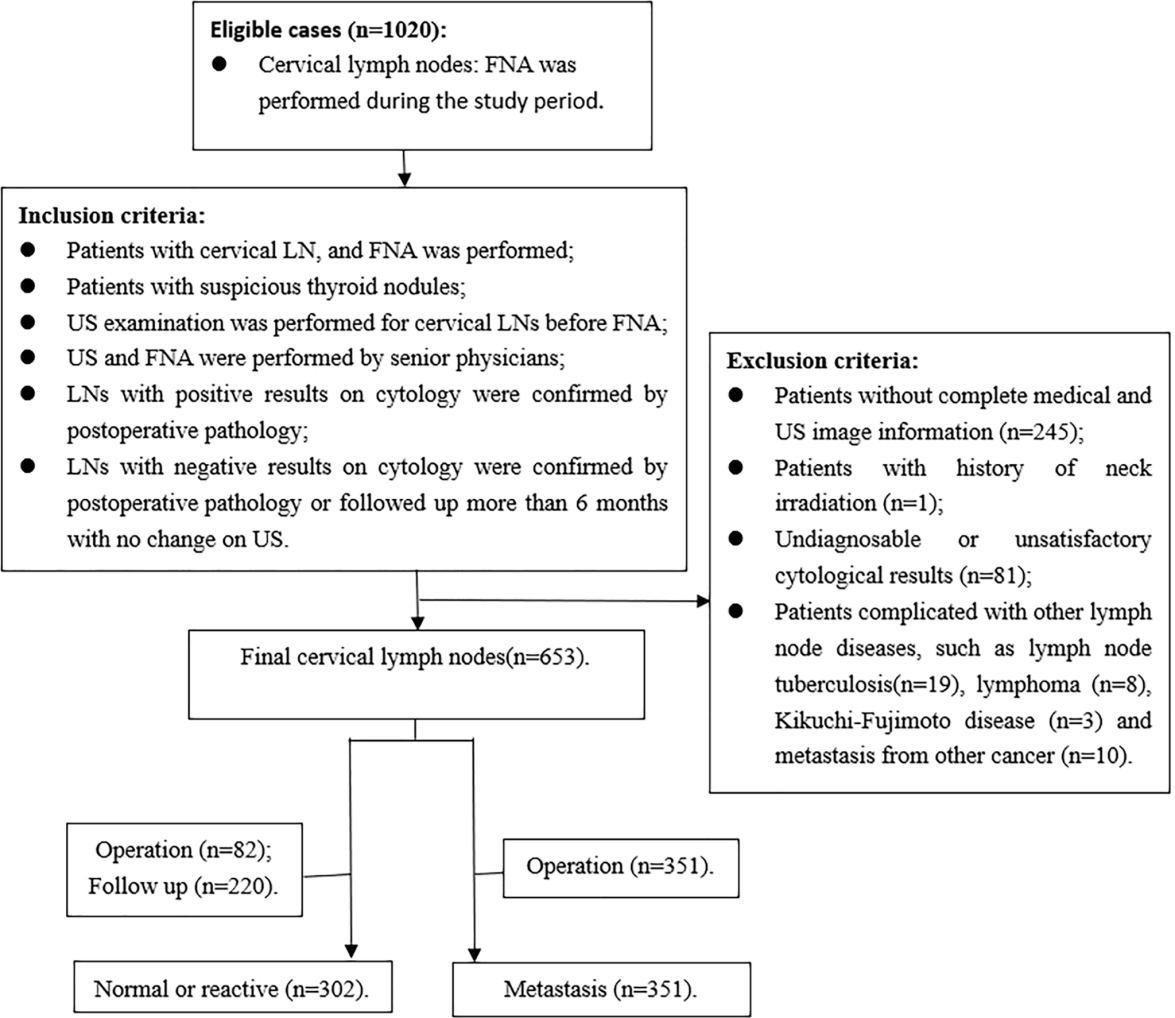
Flow chart shows selection of study population and diagnoses of lymph nodes.

The inclusion criteria were as follows: (1) FNA was performed for the patients with cervical LN; (2) patients with suspicious thyroid nodules, which showed local invasion, vertical orientation, microcalcification, and markedly hypoechoic (12); (3) US examination was performed for cervical LNs before FNA; (4) US and FNA were performed by senior physicians; (5) LNs with positive results on cytology were confirmed by postoperative pathology; (6) LNs with negative results on cytology were confirmed by postoperative pathology or followed up more than 6 months with no change on US. Exclusion criteria were as follows: (1) patients without complete medical and US image information; (2) patients with history of neck irradiation; (3) undiagnosable or unsatisfactory cytological results, including cyst fluid only, virtually acellular specimen, etc; (4) patients with other LN diseases, such as tuberculosis, lymphoma, Kikuchi-Fujimoto disease, and metastasis from medullary thyroid carcinoma or other carcinomas (**Figure 1**).

### Ultrasonography Examination

All grayscale and Doppler sonographic examinations were performed with a 4- to 13-MHz linear probe (MyLab 90; EsaoteSpA, Genoa, Italy, iU22 system, Philips, Seattle, WA, USA& Mindray Resonan7, China) by 2 radiologists with more than 10 years of experience in thyroid sonography and FNA. The focus, gain, and depth were adjusted to obtain the best image. Doppler parameters were optimized to maximize Doppler sensitivity.

During the sonographic examination, the patient was in the supine position, and the anterior area of the neck was fully exposed. The sonographic appearances of LNs were evaluated carefully, including size, long-axis diameter/short-axis diameter (L/S), shape, border, echogenicity, hilum, calcifications (macrocalcifications or microcalcifications), cystic change, and vascularity pattern. On the longitudinal section, the long axis and short axis of LNs were measured. L/S was categorized into ≤2 and >2 (13). Shape was divided into irregular and regular. Border was assessed as clear and unclear. Compared with the surrounding muscles, the cortex echogenicity was determined as hyperechoic and isoechoic or hypoechoic. Hilum, calcifications, and cystic change were defined as absent or present. Vascularity pattern was classified as hilar flow or avascular and abnormal flow. If capsular or peripheral flow and mixed flow were observed, they were regarded as abnormal flow (13).

When the patient had multiple LNs in one region, only the most suspicious LN or the largest LN was included. All images were recorded and uploaded to a picture archiving and communication system for later retrospective analysis. The grayscale and color Doppler sonographic features of the target LNs were assessed by two radiologists in consensus.

### US-FNA

FNA was performed for LNs with at least one of the following features: absence of a hilum; round shape; increased short axis; increased central vascularization; microcalcifications; partially cystic appearance; peripheral or diffusely increased vascularization; hyperechoic tissue (14). Meanwhile, our study also included patients for whom biopsy of palpable lymph nodes was requested by surgeons. US-FNA was performed by using a 23- or 25- gauge needle attached to a 5-ml syringe, and it was performed at least three times for each LN. All the smears were evaluated by an experienced cytopathologist. When the results were undiagnosable or unsatisfactory, these cases would be eliminated. If the cytological results of central or lateral LNs were positive, LN dissection of the corresponding region was performed, and pathological examination was conducted. If the cytological results were negative, only ipsilateral central LN dissection was performed. The lateral LNs were followed up, and it was regarded as negative if it had no changes in the follow-up for more than 6 months (11, 15).

### Statistical Analysis

Mann-Whitney U test, χ2, and Fisher exact tests by univariate analysis were used to analyze clinical information, grayscale, and color Doppler sonographic features. A multiple logistic regression analysis was applied to evaluate the malignancy risk for the independent features. All data were classified into two categories. We used a receiver operating characteristic (ROC) curve analysis to identify the best cut-off points for age and size.

ORs and 95% confidence intervals (CIs) of the features were calculated, and ORs were used for risk-weighting analyses. On the basis of the statistical characteristics, ORs were classified into the following groups: 1.0 to 1.1 suggested no association and was weighted for 0 point; 1.2 to 1.4, weak association, weighted for 1 point; 1.5 to 2.9, moderate association, weighted for 2 points; and 3.0 to 9.9, strong association, weighted for 3 points (16). When OR was more than 10, it indicated infinite association and was further classified into: 10 to 19.9, weighted for 4 points; 20 to 49.9, weighted for 5 points;, 50 or greater, weighted for 6 points.

According to the points corresponding to the weight of sonographic features, a total score was obtained for each LN, and a risk stratification of LNs was established, depending on the distribution of malignancy rate for each score.

In all the analyses, a p value < 0.05 was considered to indicate statistical significance. The ROC curve and the area under the receiver operating characteristic curve (AUC) were used to evaluate the discrimination ability of the risk stratification model. AUC was also used to validate the lateral and central groups. The statistical analysis was performed using SPSS version 25 (IBM Corporation, Armonk, NY, USA).

## Results

### Clinical Characteristics of Patients

A total of 576 patients with 653 LNs who underwent FNA were included in the study, with 422 females and 154 males. The mean age was 41.54± 13.15 years (age range, 13-83 years). FNA was performed for all the LNs, and 351 were metastatic LNs of PTC on cytology, which were confirmed by postoperative pathology. In addition, 302 LNs were normal or reactive on cytology. Of these LNs, 82 were confirmed by postoperative pathology, and 220 had no changes in the US follow-up for more than 6 months. The clinical characteristics of the patients are shown in **Table 1**. There were no differences in sex, side, and location between the metastatic group and the benign group. Patients in the metastatic group were younger than those in the benign group (*P*=0.003, Z=-2.984). The ROC curve was used for age, and the cutoff point was 39.5, with the AUC of 0.428 (95%CI 0.381-0.475).

### Univariate Analysis and Multivariate Logistic Regression Analysis of Ultrasound Features of LNs

The univariate analysis results of US features are shown in **Table 1**. There were significant differences in short-axis diameter, L/S, hilum, echogenicity, calcifications, cystic change, and vascularity pattern between benign and metastatic LNs (*P*<0.05), but there were no statistical differences in long-axis diameter, shape, and border (*P*>0.05). The ROC curve was used for the long- and short-axis diameters, and the cutoff points of long-axis and short-axis diameters were 11.55 and 6.25, with the AUC of 0.538 (95% CI: 0.494-0.582) and 0.575 (95% CI: 0.532-0.619), respectively.

**Table 1:** Univariate analysis of clinical characteristics and ultrasound features of lymph nodes in metastatic and benign groups.

		Metastasis	Benign	*P* value
**No. of LNs**		351	302	
**No. of patients**		307	269	
**Clinical Characteristics**				
**Sex**	Male	90 (29.3%)	64 (23.8%)	0.135
	Female	217 (70.7%)	205 (62.2%)	
**Age**		39.86 ± 12.13	43.46 ± 13.01	0.003
**Side**	Right	175 (49.9%)	130 (43.0%)	0.082
	Left	176 (51.1%)	172 (57.0%)	
**Location**	Central	57 (16.2%)	56 (18.5%)	0.438
	Lateral	294 (83.8%)	246 (81.5%)	
**Ultrasound Features**				
**Long-axis diameter**		13.08 ± 7.62	11.98 ± 6.62	0.096
	>11.55	167 (47.6%)	121 (40.1%)	0.054
	<11.55	184 (52.4%)	181 (59.9%)	
**Short-axis diameter**		6.21 ± 3.24	5.40 ± 2.42	0.001
	>6.25	135 (38.5%)	78 (25.8%)	0.001#
	<6.25	216 (61.5%)	224 (74.2%)	
**L/S**	≤2	197 (56.1%)	145 (48.0%)	0.039#
	>2	154 (43.9%)	157 (52.0%)	
**Shape**	Irregular	7 (2.0%)	5 (1.7%)	0.748
	Regular	344 (98.0%)	297 (98.3%)	
**Border**	Unsharp	4 (1.1%)	10 (3.3%)	0.063*
	Sharp	347 (98.9%)	292 (96.7%)	
**Hilum**	Absent	258 (73.5%)	147 (48.7%)	0.000#
	Present	93 (26.5%)	155 (51.3%)	
**Echogenicity**	Hyperechoic	111 (31.6%)	19 (6.3%)	0.000#
	Isoechoic or hypoechoic	240 (68.4%)	283 (93.7%)	
**Calcifications**	Present	212 (60.4%)	65 (21.5%)	0.000#
	Absent	139 (39.6%)	237 (78.5%)	
**Cystic change**	Present	53 (15.1%)	1 (0.3%)	0.000*#
	Absent	298 (84.9%)	301 (99.7%)	
**Vascularity pattern**	Abnormal flow	154 (43.9%)	34 (11.3%)	0.000#
	Hilar flow or absent flow	197 (56.1%)	268 (88.7%)	

^*^Using Fisher’s exact tests, others using chi-square (X2),# enrolled into Multivariate Logistic Regression.

The indicators with statistical significance in the univariate analysis were included in the multivariate logistic regression analysis to further evaluate the malignancy risk of the independent features. The results of multivariate logistic regression analysis are listed in **Table 2**, and it showed that L/S ≤2, absence of hilum, hyperechogenicity, calcifications, cystic change, and abnormal vascularity pattern were related to metastatic LNs (*P*<0.05), which were incorporated into the risk stratification model. The short-axis diameter was excluded because of no statistical significance (*P*>0.05).

**Table 2:** Odds ratios for the selected sonographic features by multivariate logistic regression analysis and their corresponding weighting.

	P Value	Odds Ratio	95% Confidence Interval	Corresponding weighting
**Short-axis diameter**	0.653	1.106	0.713-1.715	/
**L/S**	0.015*	1.644	1.102-2.453	2
**Hilum**	0.002*	1.894	1.265-2.835	2
**Echogenicity**	0.000*	5.375	2.936-9.842	3
**Calcifications**	0.000*	6.201	4.127-9.317	3
**Cystic change**	0.000*	71.818	9.408-548.249	6
**Vascularity pattern**	0.000*	3.811	2.331-6.230	3

^*^P Value<0.05.

### Development of a Risk Stratification Model

According to the ORs obtained from multivariate analysis, L/S ≤2 and absence of hilum were scored 2 points, hyperechogenicity, calcifications, and abnormal vascularity pattern were scored 3 points, and cystic change was scored 6 points (**Table 2**). A total score was obtained for each LN, and the malignancy rate of each score was calculated. The higher the nodule score, the higher the risk of malignancy (**Table 3**).

**Table 3:** Predictive total scores and malignancy rates of lymph nodes.

Score	No. of Malignant (n = 351)	No. of LNs (n = 653)	Malignancy Rate, %
**0**	7	69	10.14
**1**	NA	NA	NA
**2**	12	110	10.91
**3**	18	36	50
**4**	23	71	32.39
**5**	62	97	63.92
**6**	16	21	76.19
**7**	51	71	71.83
**8**	48	54	88.88
**9**	3	3	100
**10**	41	46	89.13
**11**	27	30	90.00
**12**	1	1	100
**13**	27	29	93.10
**14**	5	5	100
**15**	NA	NA	NA
**16**	3	3	100
**17**	3	3	100
**18**	NA	NA	NA
**19**	4	4	100

NA, not available.

**Figure 2:**
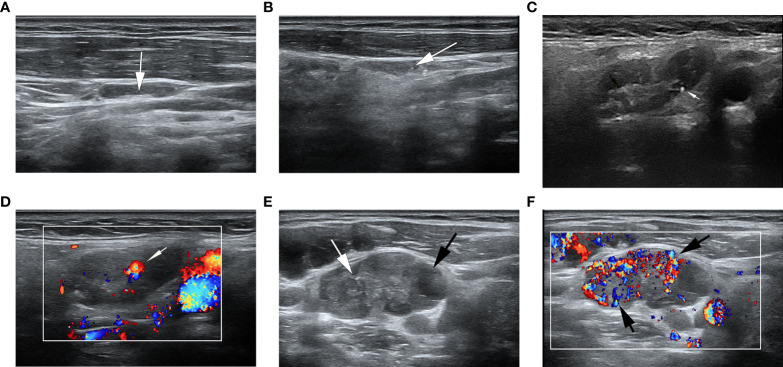
Ultrasonographic image of lymph nodes. **(A)** A metastatic lymph node in a 32-year-old man with PTC was classified as low suspicion. The lymph node was located in right level IV and measured 17.7×3.8mm, with echogenic hilum (white arrow) and L/S>2. The total score was 0. **(B)** A metastatic lymph node in a 39- year-old woman with PTC was classified as intermediate suspicion. The lymph node was located in left III level, and grayscale sonogram demonstrated that the lymph node was measured 14.4×3.5mm with microcalcifications (white arrow) and no echogenic hilum. The total score was 5. **(C, D)** A suspicious lymph node in a 63-year-old woman with PTC was classified as intermediate suspicion. The cytological result was benign, and follow-up ultrasound was performed. The lymph node was located in right II level. The total score was 3. **(C)** The grayscale ultrasonographic image showed a hypoechoic lymph node, which was measured 25.2×9.4mm, with echogenic hilum and calcification (white arrow). **(D)** Color Doppler showed that the lymph node had a hilar vascularity (white arrow). **(E, F)** A metastatic lymph node in a 33-year-old woman was classified as high suspicion. The lymph node was located in left IV level. The total score was 19. **(E)** The gray ultrasonographic image showed a hyperechoic lymph node, which was measured 28.2×14.7mm, with a L/S ≤2 shape, absence of hilum, microcalcifications (white arrow), and cystic change (black arrow). **(F)** Color Doppler showed that the lymph node had a mixed vascularity pattern (black arrow).

Then, the total score was simplified into 3 groups on the basis of malignancy rate (**Table 4**): 0-2 points, with the malignancy rate of 10.61%, related to low suspicion; 3-5 points, with the malignancy rate of 50.49%, related to intermediate suspicion; ≥6 points, with the malignancy rate of 84.81%, related to high suspicion (**Figure 2**). A risk stratification model for metastatic LNs including suggestion for clinicians was established according to the distribution of the malignancy rate (**Table 4**). The ROC curve showed that the AUC for the risk stratification model was 0.827 (95% CI 0.795-0.859). The malignancy rates of the lateral group and the central group were also calculated (**Table 4**). The AUC for the lateral group was 0.838 (95% CI 0.803-0.873), and it was 0.763 (95% CI 0.675-0.851) for the central group.

**Table 4:** Simplified risk score and risk stratification.

	Total (n=653)			Lateral group (n=540)				Central group (n=113)				
Score	No. of Malignant	No. of LNs	Malignancy Rate, %	No. of Malignant	No. of LNs	Malignancy Rate, %		No. of Malignant	No. of LNs	Malignancy Rate, %	Risk stratification	Suggestion
**0-2**	19	179	10.61	13	147		8.84	6	32	18.75	Low suspicion	Follow up
**3-5**	103	204	50.49	80	157		50.56	23	47	48.94	Intermediate suspicion	FNA
**≥6**	229	270	84.81	201	236		85.17	28	34	82.35	High suspicion	FNA or surgery

## Discussion

In recent years, the incidence of thyroid cancer has increased rapidly, and the presence or absence of LN metastases determines the surgeon’s operation method. US is used as a primary diagnostic tool to detect cervical metastatic LNs of PTC, and the evaluation indicators included site, size, boundary, hilum, matting, internal nodal echo patterns, and vascularity pattern (17). US-FNA had been commonly used to diagnose lymphadenopathy due to its safety and high accuracy (18–20).

Due to the complexity of sonographic features, several US risk stratification systems were developed to standardize the execution process of US and the diagnostic criteria. In 1992, The American College of Radiology (ACR) first launched Breast Imaging Reporting and Data System (BI-RADS), standardized stratification of the malignant risk of breast lesions. The corresponding treatment suggestions were also put forward. Subsequently, various versions of risk stratification of thyroid nodules were brought out in several countries over the years. Estimated risk of malignancy of American Thyroid Association (ATA) management guidelines in 2015 (8) and ACR Thyroid Imaging Reporting and Data System (TI-RADS) in 2017 (9) were most commonly used. The risk stratification systems have been widely recognized and applied in clinical practice worldwide. All these systems showed that the diagnosis standard could be more standardized and unified by combining with the diagnosis of imaging reporting and data system and risk stratification, which had significant clinical value, making better communication between radiologists and clinicians.

Previous studies had researched independent factors that were related with cervical metastatic LNs; however, to the best of our knowledge, there were only few studies focused on LN risk stratification on US. According to the European Thyroid Association Guidelines (14), cervical LNs in patients with thyroid cancer were classified into three groups: normal, indeterminate, and suspicious for malignancy. This LN model had been most widely used; however, it could not cover all types of LNs. The LNs with presence of hilum could not be classified when they were accompanied by the characteristics of round shape, increased short axis, or increased central vascularization. Moreover, different features had different risk of malignancy. Hyperechogenicity, calcifications, and cystic change were the most specific sonographic features to differentiate benign from metastatic nodes (13). Therefore, the weights of different malignant features should be taken into account.

Ryu et al (11) developed a cervical LN imaging reporting and data system in 2016, suggesting round, hyperechogenicity, absence of hilum, calcifications, presence of peripheral and mixed vascular patterns, and malignant real-time elastography assessment as suspicious features. Finally, according to the numbers of suspicious features, the model was classified as probably benign, low suspicion for malignancy, moderate suspicion for malignancy, high suspicion for malignancy, and highly suggestive of malignancy. This model was simple to use and included elasticity scores, but there were still some limitations. All types of LNs were included in the study, and it was not particularly for LNs in the patients with thyroid cancer. The ultrasonic characteristics of LNs of different pathological types varied greatly (21), which might reduce the accuracy of the model. In addition, the contributions of different characteristics to the risk of malignancy were also not considered, and each suspicious feature was regarded with the same weight.

Our study was to develop a risk stratification model on metastatic LNs of PTC, and the weights of different malignant indicators were also considered. Moreover, we only included cervical LNs in the patients with suspicious thyroid nodules, and other malignant and tuberculous LNs were excluded. There were no differences in sex, side, and location between metastatic and benign groups; however, patients in the metastatic group were younger than those in the benign group, with the cutoff age of 39.5. Age was considered to be a factor associated with LN metastasis. According to a meta-analysis reported by Qu (22), the risk of central LN metastasis significantly decreased in patients with age >45 years. But age was not included in multivariate analysis in our study because it was only a clinical factor instead of an ultrasonic feature.

The grayscale and color Doppler characteristics of LNs were analyzed by univariate and multivariate analysis in the metastatic and benign groups. The results showed that there were statistical differences in the features of L/S, hilum, echogenicity, calcifications, cystic change, and vascularity pattern between the two groups. It implied that the metastatic LNs of PTC tended to have a feature of L/S ≤2, absence of hilum, hyperechogenicity, calcifications, cystic change, and abnormal vascularity pattern, which were similar to the results reported in previous studies (23, 24).

The short-axis diameter was more significant than the long-axis diameter for evaluating LNs, and the thresholds of the short-axis diameter were 0.5, 0.8 and 1 cm according to different regions (25). But specific cutoff points were difficult to find because of the overlap between metastasis and non-metastasis (26). In our study, the short-axis diameter > 6.25 was different between metastatic and benign LNs according to the ROC curve, but it was not the independent risk factor. Absence of hilum was commonly seen in malignant LNs, but some benign LNs could also show the sign, and absence of hilum did not always suggest malignancy (26). Normal LNs had an oval shape, with L/S >2. Conversely, malignant LNs tended to have a rounded morphology, with L/S ≤2 (23). Hyperechogenicity, calcifications, and cystic change were specific features of metastatic LNs, with ORs of 5.375, 6.201, and 71.818, highly indicating malignancy. However, calcifications were also found in 65 benign LNs, and the specificity was lower than that in previous studies (14, 27). The indicator of calcification included both microcalcification and macrocalcification in our study. Microcalcification was a specific feature of metastatic LNs of PTC, and macrocalcification was common in granulomatous disease (28). All the LNs underwent FNA in our study, and the LNs with negative FNA results were followed up by US; however, there was a certain false negative rate of FNA. Meanwhile, core needle biopsy was not performed. Some benign diseases of LNs with macrocalcifications, such as latent tuberculosis, might not be diagnosed by FNA, and these cases might be also included in our study. In addition, punctate echogenic foci in LNs could be mistaken for microcalcifications due to the retrospective nature of this study. Color Doppler revealed that hilar flow was a characteristic for benign nodes, while peripheral and mixed flow were regarded as characteristics for malignant nodes (29, 30). In our study, abnormal flow was also a risk factor for metastatic LNs.

According to ORs obtained by multivariate logistic regression analysis in our study, we assigned weights to statistically significant features from 2 to 6 points, which could reflect the risk of different features. The assignment of this classification was based on a previous study and clinical practice (16). Cystic change had the highest weight (OR=71.818) and was assigned 6 points, standing for the highest risk of malignancy. Hyperechogenicity, calcifications and abnormal flow were assigned 3 points (OR=5.375, 6.201 and 3.811), while L/S ≤ 2 and absence of hilum were assigned 2 points (OR=1.644 and 1.894).

Finally, we formulated the risk stratification model based on the distribution of the total score and the malignancy rate. The AUC for the model was 0.827, which indicated a good discrimination ability. We made suggestions for each category according to the common clinical management. For 0-2 points, there was no malignant feature on US, or one of L/S ≤ 2 or absence of hilum. Most metastatic LNs had no visible hilum or L/S ≤ 2, but the features taken as single criteria were not specific enough to suspect malignancy (27). According to the total malignancy rate of 10.61%, it was classified as low suspicious malignancy (recommended follow-up 12-24 months) (8). The malignancy rate of central LNs was higher than that of the lateral group in the low suspicious malignancy group (18.75% vs 8.84%);, however, prophylactic central LN dissection was accepted as a standard treatment (31). Therefore, treatment strategies would not be affected. Points of 3-5 showed one feature of hyperechogenicity, calcifications and abnormal flow, with or without one of L/S ≤ 2 and absence of hilum. L/S ≤ 2 concomitant with absence of hilum could be assigned 4 points, which was also classified to this group. According to a higher malignancy rate, it was classified as moderate suspicious. It was difficult to differentiate benign from metastatic LNs by US, and FNA should be performed. The overall malignancy rate of 6 points or more was 84.81%, which was highly suspicious malignancy (71.83%~100%). Cystic change was assigned a score of 6, with the highest OR and specificity, and was directly included in this group. FNA or surgical treatment was suggested. If the cytological result was positive, LN dissection was required. If the cytological result was negative, repeated FNA or closed follow-up was recommended. Previous studies suggested that there were differences in the sonographic features of lateral and central LNs in PTC (32). In our study, the diagnostic values of the risk stratification model in the lateral and central regions were verified by the AUC, and the AUC for the lateral group was 0.838, and it was 0.763 for the central group, indicating a good diagnostic value for both. However, due to the limited sample size, the test and verification groups were not separated, and internal and external validations were not performed.

There were several limitations in this study: 1. Selection bias was inevitable due to the retrospective nature of this study. All cases were selected from patients undergoing FNA rather than the general population. Some benign LNs without FNA were followed up, and these LNs were not included; 2. False negative results of FNA could not be ignored because not all patients with benign cytological results were confirmed by postoperative pathology; 3. FNA-thyroglobulin, which could increase the diagnostic accuracy of metastatic LNs, had not been tested; 4. The sample size was relatively small, and data were collected only in a single center. A large sample multi-center study, as well as internal or external validation, should be further performed; 5. Different subtype of PTC might affect the ultrasonic appearance and risk stratification of LN;, however, these subtypes were not statistically analyzed in our study.

## Conclusions

The features of L/S ≤2, absence of hilum, hyperechogenicity, calcifications, cystic change, and abnormal vascularity pattern were independent risk factors of metastatic LNs of PTC. Our newly established risk stratification model can effectively evaluate metastatic LNs in the patients with suspicious thyroid nodules, and a new strategy selection was supplied.

## Data Availability Statement

The raw data supporting the conclusions of this article will be made available by the authors, without undue reservation.

## Ethics Statement 

This study was approved by the Ethics Committee of our hospital, and formal consent is not required for it is a retrospective study.

## Author Contributions

All authors contributed to the study conception and design. Material preparation, data collection, and analysis were performed by XN and SX. The first draft of the manuscript was written by XN and all authors commented on previous versions of the manuscript. All authors read and approved the final manuscript. All authors contributed to the article and approved the submitted version.

## Funding

This work was supported by the National Natural Science Foundation of China (NO:82071923).

## Author Disclaimer

The funder(s) conceived of the present idea of the study, but had no role in study design, data analysis, data interpretation or in the decision to submit the manuscript for publication.

## Conflict of Interest

The authors declare that the research was conducted in the absence of any commercial or financial relationships that could be construed as a potential conflict of interest.

## Publisher’s Note

All claims expressed in this article are solely those of the authors and do not necessarily represent those of their affiliated organizations, or those of the publisher, the editors and the reviewers. Any product that may be evaluated in this article, or claim that may be made by its manufacturer, is not guaranteed or endorsed by the publisher.

## Abbreviations

PTC, Papillary thyroid carcinoma; LN, Lymph node, US, Ultrasonography; FNA, Fine-needle aspiration; L/S, Long-axis diameter/short-axis diameter; ROC, Receiver operating characteristic; OR, Odds ratio; CI, Confidence intervals; AUC, Area under the receiver operating characteristic curve; ACR, American College of Radiology; BI-RADS, Breast Imaging Reporting and Data System; ATA, American Thyroid Association; TI-RADS, Thyroid Imaging Reporting and Data System.
